# Urban soil compaction reduces cicada diversity

**DOI:** 10.1186/s40851-015-0022-3

**Published:** 2015-08-01

**Authors:** Minoru Moriyama, Hideharu Numata

**Affiliations:** Graduate School of Science, Osaka City University, Osaka, 558-8585 Japan; National Institute of Advanced Industrial Science and Technology (AIST), Tsukuba, 305-8566 Japan; Graduate School of Science, Kyoto University, Kyoto, 606-8502 Japan

**Keywords:** Biodiversity loss, Conservation, Community structure, Soil hardness, Urban landscape

## Abstract

**Introduction:**

Urbanization converts animal habitats into globally homogeneous environments. Consequently, urban communities have low diversity and are often dominated by a few species. However, proximate environmental factor(s) causing community degradation have rarely been identified among diverse and co-varying urban parameters.

**Results:**

The present study addresses the recent loss of cicada diversity in Osaka, Japan, where cicada communities are overwhelmed by a single species, *Cryptotympana facialis*. A field survey across an urban-forest gradient revealed that the trend of decreasing cicada diversity toward the urban core was mostly associated with the soil hardness among the environmental variables examined. Simultaneously, the proportion of *C. facialis* increased with soil hardness, although this effect was partially mitigated in forest patches. Newly hatched nymphs of *C. facialis* exhibited greater burrowing ability than that in other native species.

**Conclusions:**

These findings identify soil compaction due to urbanization as a possible cause of cicada diversity loss, as it impedes the passage of nymphs to underground nests. This impact of urban soil compaction may influence ecosystem functioning of soil-dwelling arthropods and their trophically associated animals.

**Electronic supplementary material:**

The online version of this article (doi:10.1186/s40851-015-0022-3) contains supplementary material, which is available to authorized users.

## Introduction

Urbanization brings about radical changes in animal habitats, contributes to the extirpation of local populations and community collapse, and consequently disrupts ecosystem function and service delivery [9, 13, 55]. Understanding urban degradation of biotic and abiotic community processes has become an increasingly urgent conservation concern [30, 36].

It is generally accepted that animal diversity decreases toward urban cores compared to rural surroundings [11, 14, 38]. Simultaneously, urban areas are usually dominated by a limited number of species, which are referred to as “urban exploiters” [4, 37]. Urban landscapes are characterized by extensive habitat fragmentation, a high proportion of impervious ground surfaces, compacted soil, locally hot and dry climatic conditions, and elevated pollution levels [13, 36, 46]. It is difficult to use correlative analyses to discriminate a proximate factor for community change from co-varying parameters because diverse environmental parameters interact and vary with urbanization processes [49]. Moreover, responses to urbanization are highly species- or community-specific [10, 32, 53, 61]. Thus, studies aiming to identify a causal process responsible for biodiversity loss should be based on the life cycle traits and physiological or behavioral characteristics of individual species.

Until now, research on urban ecology has been developed largely for vertebrate species e.g., birds and mammals, and arthropods have received less attention in spite of their advantages regarding ethical and experimental practicability [30, 34]. Cicadas are a conspicuous insect group, especially in warm temperate and tropical zones, due to their large bodies and species-specific calling songs [2, 15, 43]. Because a change in cicada communities can easily be recognized by ordinary citizens as an acoustic change, it is an important index of biodiversity. In Japan, cicada communities have experienced substantial changes, mainly in urbanized areas [45]. A striking instance is that in Osaka City—during the extensive urban growth in the last few decades of the 20th century, the cicada community became overwhelmed by a single native cicada, *Cryptotympana facialis*, which had previously comprised a minor population [40, 45, 59]. It has brought new social problems such as loud noise of calling songs and destruction of optical fiber cables [8].

In the present study, we investigated the influence of environmental parameters on cicada community structures along an urban-forest gradient in Osaka Prefecture, with a particular focus on urban soil compaction. While cicada nymphs grow on the underground roots of trees, adults and their eggs spend all their time on aboveground parts of trees [15, 43]. Hence, newly hatched nymphs confront the critical challenge of passing through the ground surface barrier and reaching suitable roots in a brief time [19, 23, 39]. Thus, we hypothesized that soil compaction in urban areas might be a proximate cause of diversity loss in the cicada community. We therefore compared burrowing ability among nymphs of native cicada species under standardized experimental conditions in addition to field observation of cicada diversity. The results suggested that degradation of the cicada community resulting from *C. facialis* domination had been administered by urban soil compaction.

## Materials and methods

### Study sites

Osaka City is the most economically developed city in western Japan. It is located in the center of Osaka Prefecture, and surrounded by several suburban cities, including Sakai, Higashi-Osaka, and Mino. The outline of the prefecture is largely formed by mountain forest. We selected 13 sampling sites along this urban-forest gradient (Additional file 1: Table S1). Each site was assigned to one of three patch categories: a small park (0.49 − 3.74 ha), large park (25.5 − 106.7 ha), or continuous forest zone. Three to six sampling plots were defined in each site, except for small urban parks, in which only one plot was defined. The boundaries of each plot (at least two sides of a quadrangle) were defined by pavements, trails, steep slopes, or other apparent discontinuity of vegetation. The other sides were arbitrarily determined, and the area of each plot was 152.3 m^2^ on average (35.5 − 415.3 m^2^).

### Collection of cicada exuviae

By collecting exuviae of final instar nymphs, we recorded the number and species of cicadas that had emerged from the sampling plots, i.e. *C. facialis*, *Graptopsaltria nigrofuscata*, *Meimuna opalifera*, *Platypleura kaempferi*, *Hyalessa maculaticollis*, and *Tanna japonensis*. Exuviae of these cicadas are conspicuous, steady for a long time, and have often used for community assessments (e.g. [45, 59]). Before the emergence period of adult cicadas, exuviae remaining from the previous year were removed. We visited each site to collect exuviae at least two times between 9 August and 25 September 2008. At each visit, we thoroughly rummaged through the sampling plot for all exuviae, some of which had fallen to the ground, and the others stayed on tree branches or trunks at heights of up to several meters. Thus, differences in visibility of exuviae among sites seemed to have negligible effects on results. A minor portion of exuviae may have been blown off or grabbed by a passerby in the sampling intervals. Nevertheless, we found a considerable number of exuviae at every site in consistent with auditory species information. Therefore, it is presumed that most exuviae were collected with the least sampling bias for species among sites. The species of the exuviae were identified according to Hayashi and Saisho [15]. The diversity of the cicada community was evaluated using Shannon’s diversity index *H* [3].

### Environmental variables

Table 1 summarizes the environmental parameters addressed in the present study. The proportion of the impervious surface, i.e. pavement or buildings, was calculated from satellite images. These images were obtained from Google Earth ver. 7.1.1.1888, 2013, and processed using Adobe Photoshop Professional ver. 6.0 (Adobe, San Jose, CA). To measure the degree of soil compaction at the ground surface, a Yamanaka-type penetrometer No. 351 (Fujiwara Scientific, Tokyo, Japan) was used [58], and thirty measurements were performed for each plot at the time of exuvia collection. Soil hardness can be modified by hydration state. However, precipitations vary with location and time, and we were unable to know its exact amount at each sampling site. Therefore, to avoid transient effects of precipitation, we measured soil hardness after at least two consecutive sunny days. In some of the sampling plots, we collected 15 soil core samples (100 mL) at a depth of 0–50 mm for each. These soil samples were dried at 110 °C for 12–24 h, and weighed, to determine dry bulk density and water content (Additional file 2: Table S2). The percentage of canopy cover of a plot was calculated from satellite images as described above for impervious areas. We counted all trees, except for shrubs, regardless of their width, and identified 21 tree species across the sampling sites. The most prevalent tree species were *Zelkova serrata*, *Prunus* trees (*P. yedoensis* and *P. jamasakura*), *Cinnamomum camphora*, and *Ginkgo biloba*. These species comprised 64.9 % of the total tree count. Diversity of trees was represented by Shannon’s index.

**Table 1:** Environmental variables included in the model analyses for cicada community structures

Variable	Range	Description
Patch type	3 levels	Small park, large park, forest
Imperviousness	12.7–91.9	Percentage of impervious area in 1-km radius
Altitude^a^	3–319	Altitude at the site (m)
Slope	0.0–19.1	Slope of the ground (°)
Soil hardness	7.3–27.8	Index of Yamanaka's soil hardness meter (mm)
Canopy	15.6–100	Percentage of canopy coverage in 30-m radius
Tree diversity	0.0–3.1	Shannon’s index (*H*)
Tree density	2.2–20.0	Number of trees per 100 m^2^

^a^Variable excluded in multi-variable analyses

### Model analysis

We evaluated the effects of environmental parameters on cicada community structures under a generalized linear mixed model (GLMM) framework. Diversity indices (square-rooted) and proportions of a given species were fitted to a model that assumed a Gaussian or binomial error structure, with an identity or logit link function, respectively. In the latter variable, individual random effects were taken into account to avoid overdispersion. Model comparisons were performed by a chi-square test of deviance reduction.

We performed single variable analyses using each environmental variable. Quadratic terms were also evaluated in these models, and used in the further analyses if their effects were significant. In multi-variable analyses, all possible two-way interactions were considered. Some explanatory variables showed high correlation coefficients (Additional file 3: Table S3). To limit the effects of multicollinearity, the models omitted the altitude variable, whose variance inflation factor exceeded 10. We selected a parsimonious model based on the Akaike’s information criterion and then a stepwise backward procedure [7]. Finally, the levels in a factorial variable were merged when it was not significantly different from the original model. All statistical analyses were conducted using R 3.1.2 [48].

### Burrowing experiments

Adult females of four species, i.e. *C. facialis*, *G. nigrofuscata*, *M. opalifera*, and *P. kaempferi*, were collected in Osaka Prefecture between July and September in 2006–2007. The methods of egg collection and maintenance have been described in detail [40, 42]. Captured adult females were individually caged in a plastic pot (9 cm diameter, 12 cm depth), and allowed to lay their eggs into squared *Albizia* timber sticks (6 × 6 × 130 mm). These sticks were kept outdoors in a net hung on the tree branch at the campus of Osaka City University. Approximately one month before completion of embryonic development, sticks were transferred to a laboratory at 25.0 ± 1.0 °C under 16 h light and 8 h dark cycles, and hydrated at intervals of 2–5 days to induce hatching [39]. Nymphs leaving the sticks were used for burrowing experiments described below within 30 min.

The soil was collected on the campus of Osaka City University (Additional file 2: Table S2), passed through a 5-mm-mesh sieve, and dehydrated at 110 °C for 12–24 h. A grading analysis revealed that the soil type was sand with median grain diameter of 354 μm and coefficient of uniformity of 6.13. Burrowing arenas were created with cylindrical polystyrene containers (150 mm diameter, 90 mm height) filled with a mixture of 1200 g dried soil and 120 g distilled water (9.1 %), unless otherwise noted. The soil was compressed to final heights of 50, 55, 60, and 65 mm, to obtain four decreasing levels of soil compactness, 1.36, 1.23, 1.13, and 1.04 g/ml, respectively. In further experiments, the water content was increased to 150 g (11.1 %), or the soil was mixed with vermiculite (Nittai, Osaka, Japan). The compositions of the dried soil, vermiculite and distilled water were 880, 136, and 120 g (dry mass content of vermiculite was 13.4 %), or 320, 374, and 120 g (53.9 %), respectively. These mixtures had approximately the same volume as the 1200 g soil containing 120 g water before compression. In these experiments, the soil mixture was compressed to a height of 55 mm.

Ten nymphs were released on one of these burrowing arenas. The arenas were kept at 25.0 °C, and closed with a lid to prevent desiccation except during observation. Nymphs usually tried to enter the soil from small cracks on the surface. In well-compacted soil with flat surface, however, we often found newly-made tiny holes after nymphs had entered the soil. In the present study, nymphs that disappeared from the soil surface were considered to have burrowed. Although we recorded the number of burrowed nymphs every 15–30 min for 3 h, the scores at 60 min were compared among species as an ecologically relevant scale, because the previous study showed that no nymphs survive on the ground for more than 1 h in the field [39]. The effects of species and soil conditions on burrowing rates were examined using GLMM assuming a binomial error structure with a logit link function, considering individual variation as a random effect. When the species term had a significant effect, we performed pairwise comparisons with a Bonferroni adjustment.

## Results

### Cicada community change across the urban-forest gradient

**Fig. 1:**
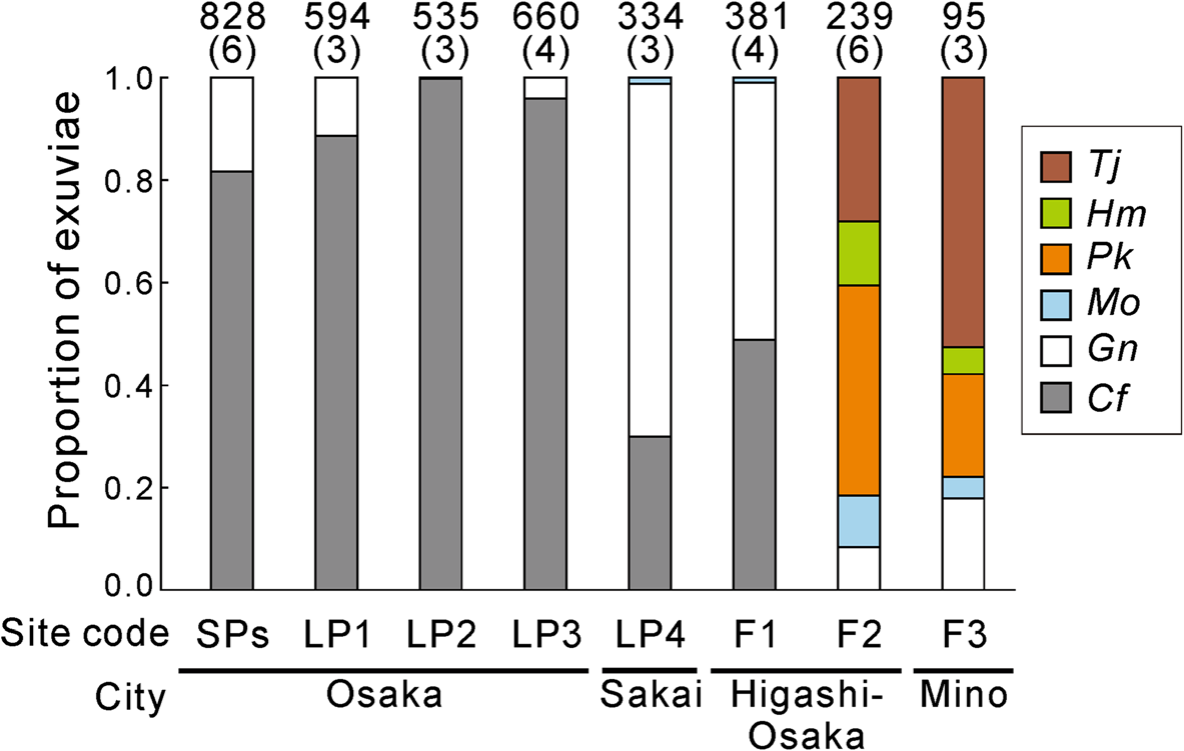
Species compositions of cicada communities across the urban-forest gradient in Osaka and surrounding cities. Site codes that indicate their patch types are given; SP: small park, LP: large park, F: forest. Six small parks were merged into SPs. The numbers above the columns indicate the number of collected exuviae (upper) and sampling plots (lower in parentheses) at the site. Species abbreviations; *Cf*: *Cryptotympana facialis*, *Gn*: *Graptopsaltria nigrofuscata*, *Mo*: *Meimuna opalifera*, *Pk*: *Platypleura kaempferi, Hm*: *Hyalessa maculaticollis*, *Tj*: *Tanna japonensis*

From 13 sampling sites across the urban-forest gradient, we collected a total of 3666 exuviae of six species of cicadas. Species richness was extremely poor in both the small parks and large parks in Osaka City due to domination by *C. facialis* (Fig. 1). The only species other than *C. facialis* found in Osaka City was *G. nigrofuscata*, and there were no exuviae of *P. kaempferi* or *M. opalifera*, which were formerly common in this city [45]. In one suburban park (LP4) and one forest fringe site (F1), *G. nigrofuscata* comprised a majority, although *C. facialis* was still numerous. Inside forest areas (F2 and F3), however, the five species other than *C. facialis* often coexisted in the same plot.

Despite the general trend across the urban-forest gradient, variations in species composition within a site were not negligible. Then, we combined data from all sampling plots, and performed model analyses using the environmental parameters listed in Table 1. Some variables used in this study varied with urbanization, and showed high correlation with each other (Additional file 3: Table S3). For example, imperviousness of the ground surface, the most relevant urbanization indicator, was positively correlated with soil hardness, but negatively correlated with altitude, slope, and canopy coverage. These trends seemed to reflect the fact that urbanization has progressed in low, flat regions, and is characterized by open habitats and compacted soil.

**Fig. 2:**
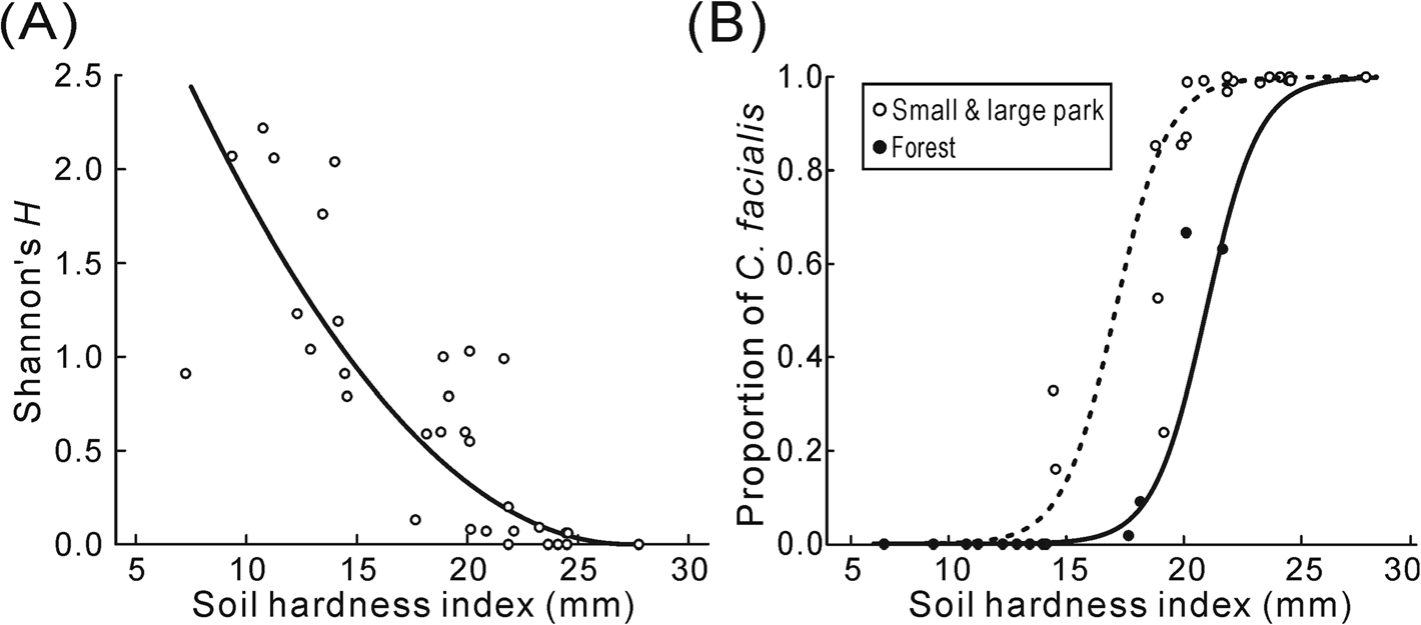
Effects of soil hardness on cicada community. **a** Relationship of soil hardness to cicada diversity with a fitted curve predicted by the model (see Table 3). **b** Relationship of soil hardness to proportion of *Cryptotympana facialis* with fitted curves for small and large parks (dashed line) and forest (solid line) patch types

When we analyzed Shannon’s diversity index as a simple representative value of species richness and evenness, the index was significantly associated with every environmental variable except the tree density in single-variable models (Table 2). In particular, diversity tended to decline at sites characterized by high imperviousness, low altitude, and high soil hardness. The multi-variable model showed that soil hardness alone was the best predictor of cicada diversity (Table 3, Fig. 2A). The proportion of *C. facialis* and the diversity index showed similar associations with environmental predictors in single-variable analyses, except that the directions of their associations with these predictors were opposite (Table 2). The multi-variable model for the *C. facialis* proportion, which contained variables for soil hardness and patch type, was most parsimonious after the small park and large park levels were merged as the latter variable (Table 3). In this model, soil hardness had three-times greater influence than patch type, as judged by the proportion of deviance explained (66.1 vs 21.9). This indicated that the proportion of *C. facialis* in forest regions tended to be lower at a given soil hardness than that in park sites (Fig. 2B). Taken together, these models implied that a reduction in cicada diversity mainly attributed to an increase in the *C. facialis* population was associated with urban soil compaction.

**Table 2:** Single-variable analyses for cicada diversity index and proportion of *Cryptotympana facialis*

	Shannon's *H* (square root)		Proportion of *C. facialis*	
Variable	Coefficient ± SE	*P*-value	Coefficient ± SE	*P*-value
Patch type (SP, F)^a^	**−**0.780 ± 0.174	3.37e^**−**7^	9.08 ± 1.72	4.91e^**−**8^
(LP,F)^a^	**−**0.642 ± 0.138		9.87 ± 2.01	
Imperviousness	**−**0.013 ± 0.002	1.25e^**−**11^	0.187 ± 0.031	9.99e^**−**10^
Altitude	3.0e^**−**3^ ± 0.5e^−3^	3.42e^**−**10^	**−**0.083 ± 0.021	7.85e^**−**5^
Slope	0.050 ± 0.012	2.23e^**−**5^	**−**0.785 ± 0.195	5.56e^**−**5^
Soil hardness	**−**0.079 ± 0.009	2.43e^**−**17^	1.14 ± 0.14	2.08e^**−**15^
Canopy	0.013 ± 0.003	7.52e^**−**7^	**−**0.165 ± 0.032	2.87e^**−**7^
Tree density	0.024 ± 0.018	0.199	**−**0.246 ± 0.277	0.375
Tree diversity	**−**0.229 ± 0.104	0.028	3.28 ± 1.51	0.029

^a^The patch-type effects of small park (SP) or large park (LP) against forest (F) are indicated

**Table 3:** Parsimonious models for cicada diversity, abundance, and the proportion of *Cryptotympana facialis*

Component	Coefficient ± SE	*P*-value	Residual/null deviance
Shannon's *H* (square root)			2.15 / 7.31
(intercept)	2.15 ± 0.18		
Soil hardness	−0.079 ± 0.009	2.43e^−17^	
Proportion of *Cryptotympana facialis*			67.1/143.7^b^
(intercept)	−18.7 ± 2.18		
Soil hardness	0.89 ± 0.11	3.97e^−16^	
Patch type (small & large park, forest)^a^	3.44 ± 0.74	2.57e^−06^	

^a^The levels in small and large parks were merged, and its patch-type effects against forest were indicated

^b^The null model contained a random effect term

### Comparison of burrowing ability

**Fig. 3:**
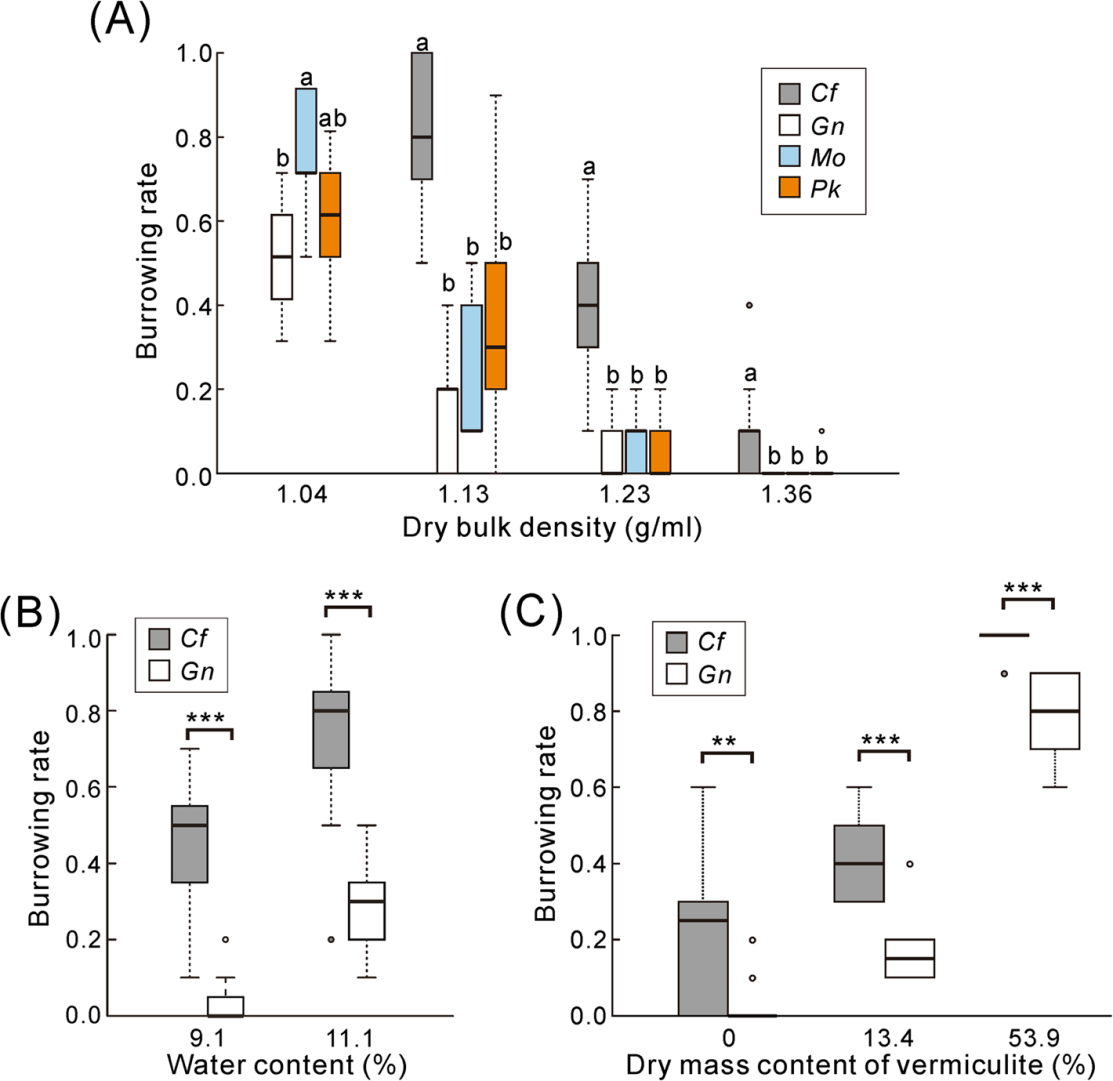
Comparisons of burrowing ability in newly hatched nymphs. **a** A Tukey boxplot for the effect of soil dry bulk density. No test was performed for *Cryptotympana facialis* at the lowest soil density. Boxes marked with a same letter did not significantly different (*P* > 0.05, GLMM followed with post-hoc multiple comparison). Each treatment consisted of 5–13 replicates with 10 nymphs each. **b** A Tukey boxplot for the effect of water content. Each treatment consisted of 7–11 replicates. **c** A Tukey boxplot for the effect of dry mass content of vermiculite. Soil porosity was controlled by mixing various proportions of vermiculite. Each treatment consisted of 10 replicates. In (**b**) and (**c**) Significant differences between species are indicated by asterisks (**P < 0.01, ***P < 0.001, GLMM). Species abbreviations; *Cf*: *Cryptotympana facialis*, *Gn*: *Graptopsaltria nigrofuscata*, *Mo*: *Meimuna opalifera*, *Pk*: *Platypleura kaempferi*

To examine whether there was a causal link between the soil status and community structure of cicadas, we compared the burrowing ability of newly hatched nymphs under laboratory conditions. The soil used in this experiment was collected on the campus of Osaka City University, in which the soil density was 1.35 ± 0.10 g/cm^3^ (mean ± SD, *n* = 15), the soil water content was 8.82 ± 2.89 %, and only *C. facialis* was found (Additional file 2: Table S2). When nymphs were released on the soil with four levels of compaction, higher soil density was associated with lower burrowing success in all species (Fig. 3A). Although the species and soil density had significant effects on burrowing rates (*P* < 0.001, GLMM), their interaction did not (*P* > 0.05). There was a clear difference in burrowing rate between *C. facialis* and the other three species. At the highest soil density, the closest condition to that of the soil collection site, no nymphs other than *C. facialis* burrowed, except for one individual of *P. kaempferi*.

Compared to urban soil, forest soil contains more heterogeneous materials with a porous nature and higher water content. When the water content was elevated from 9.1 to 11.1 %, burrowing rate increased in both *C. facialis* and *G. nigrofuscata* (Fig. 3B). Species and water content had significant effects (*P* < 0.001, GLMM), but there was no significant interaction effect (*P* > 0.05), indicating that water content affected burrowing success similarly in these two species. Finally, the soil was mixed with vermiculite to mimic litter-containing porous soil. Burrowing rate increased with vermiculite content in both species (Fig. 3C). Like water content, the species and vermiculite content had significant effects (*P* < 0.001), but their interaction did not (*P* > 0.05). Therefore, in all soil conditions tested, the burrowing rate of *C. facialis* nymphs never fell below those of the other native cicada species.

## Discussion

The present study revealed the possible involvement of soil hardness in the species distribution of cicadas. It has been shown that geographical distributions of cicadas are influenced by several abiotic factors, such as temperature [16, 42, 51, 60], humidity [41], and light [65]. Several authors have pointed out the possible relevance of soil properties to species distribution or abundance of cicadas, although their causal relationships have not been verified [5, 12, 18, 26]. For soil-dwelling arthropods, such as ants [17, 21], digger wasps [33, 47, 54], tiger beetles [6], and larvae of May beetles [25], soil characteristics play an important role in determining species-specific distribution patterns. Especially, soil hardness is strongly related to underground activities, and therefore an appropriate digging ability appears to have been selected according to the requirements of the particular habitat [33, 47, 54]. Cicadas spend much time underground during their nymphal period [23]. Once settled in underground nests, they are less mobile, and have relatively low mortality until emergence from the soil [19, 23]. However, considerable mortality occurs at the transition stage, in which tiny nymphs just after hatching from tree branches try to penetrate the ground surface to seek a suitable rootlet. Nymphs on the ground suffer extremely high risk of predation and desiccation unless they escape into the soil [19, 22, 23, 39]. The present study revealed that the distribution pattern of *C. facialis* associated with hard soil surface is consistent with its higher burrowing ability in the newly hatched stage. These findings suggest that soil hardness at the ground surface is a critical abiotic factor affecting the success of burrowing, and consequent colonization of cicadas.

We found that soil hardness was elevated in parallel with urbanization across the study sites. Soil in urbanized areas has generally been subjected to extensive tillage and compression during construction processes, repeated vehicle and foot traffic, loss of plant litter input, and changes in hydrology [20, 29, 46]. Consequently, urban soil shows non-porous compaction, low water content, low organic matter content, low water retention capacity, high pH, and high metal content. Such soil modifications can become a driving force in altering the soil arthropod community [35, 44, 52]. In the mid-20th century, *G. nigrofuscata*, *M. opalifera*, and *P. kaempferi* accounted for large portions, whereas the population of *C. facialis* accounted for a minor portion, of the cicada community in Osaka City [45]. In the present survey, the diversity of the community in this region was extremely poor due to exclusive domination by *C. facialis*. During the late 20th century, rapid and extensive urbanization proceeded in Osaka, and seems to have rendered the soil compacted and hardened to states that are unusual in nature. The resultant urban soil is likely to have favored colonization of *C. facialis* due to its higher burrowing ability. We conclude, therefore, that soil compaction derived from extensive urbanization may have served as a proximate factor accounting for the time-course of the drastic change in the cicada community.

Two formerly common species, *P. kaempferi* and *M. opalifera*, were not found in Osaka City in the present survey, suggesting their possible local extirpation. The other previously common native species, *G. nigrofuscata*, barely persisted in this area, and populated more semi-urban areas than the former two species, although all three of these species were shown to have similar burrowing ability. *G. nigrofuscata* is the most prevalent lowland species in the main island of Japan [15], and formed the largest population in Osaka City before the extensive urbanization [45]. Therefore, the higher ability of *G. nigrofuscata* to exploit lowland environments, except with regard to soil hardness, may be responsible for the current distribution patterns. In other words, soil compaction may place a similar burden across these species, and consequently species having smaller population sizes may be more susceptible to urbanization-driven extirpation.

The present findings raise the question of why *C. facialis*, in spite of its higher burrowing ability, accounted for only a minor portion of the cicada community before the extensive urbanization. Nymphs of *C. facialis* exhibited better burrowing ability in all soil conditions tested here, which included conditions mimicking soft, hydrated, or highly porous forest soil (Additional file 2: Table S2). Thus, it is unlikely that soil properties in less urbanized areas adversely affect the burrowing success of *C. facialis*. One possibility is that population growth of *C. facialis* had previously been limited by some environmental factor(s) negatively correlated with urbanization. In urban areas, air temperature is locally increased due to heat island effects, whereas humidity is decreased. We previously demonstrated that embryonic development of *C. facialis* requires greater thermal accumulation, and that the recent warming in Osaka was a prerequisite for its colonization in this area [42]. Moreover, eggs of *C. facialis* show higher desiccation tolerance than those of *G. nigrofuscata* [41]. These developmental and physiological characteristics of *C. facialis* that are favored by a hot, dry climate may have prevented its population growth before the advent of the urban climate.

Another possibility is that interspecific competition had suppressed *C. facialis* population growth. Cicadas usually exploit a wide range of plant species for feeding and oviposition [27, 62], and thus several species often share the same host tree. In the present study, exuviae of *C. facialis* were often found on the most prevalent tree species, such as *Z. serrata*, *P. yedoensis*, *C. camphora*, and *G. biloba*. These tree species are known to be exploited by the formerly common native species [24]. Adult cicadas are well known to show segregation patterns with sympatric competitors in terms of time [5, 56], microhabitat conditions [50, 51, 57], and acoustic characteristics [50, 56]. Crowding in the underground nymphal stage also has adverse effects [22, 63]. The observed negative influence of the forest patch on *C. facialis* in the model analyses may reflect inappropriate climate conditions and/or elevated competition pressure by the other species that came from surrounding source populations.

## Conclusions

The present study demonstrated that the decline in cicada diversity due to *C. facialis* domination across an urban-forest gradient is best predicted by soil hardness. We have also showed that the burrowing ability of newly hatched nymphs is remarkably higher in *C. facialis* than in other native species, which have decreased with urbanization. These consistent findings highlighted soil compaction as a proximate cause of cicada community degradation. The impact on community structures demonstrated here is predicted to be shared by diverse taxonomic groups across cities, because of the global homogeneity of urban environments [4], and because of the common importance of soil hardness among soil-dwelling arthropods [33, 47, 54]. Identification of proximate factors may also contribute to designing feasible and effective actions for biodiversity conservation [55]. Cicadas play a substantial role in ecosystem function, such as nutrient flux from underground to aboveground [1, 5, 64, 66], and are important resources for vertebrate predators [28, 31]. Therefore, maintaining a variety of cicada species with different body sizes and emergence periods is expected to support diverse animals.

## Additional files

Additional file 1: Table S1. Summary of study sites for collection of cicada exuviae. Additional file 2: Table S2. Soil properties at representative sampling plots. Additional file 3: Table S3. Correlation table for the explanatory variables.
