# Supplementary material for: Urban soil compaction reduces cicada diversity
Source: Zoological Lett. 2015 Aug 1;1:19. doi: 10.1186/s40851-015-0022-3 (PMC4657352; doi:10.1186/s40851-015-0022-3)
Supplement: Additional file 1: Table S1. — Summary of study sites for collection of cicada exuviae. [file 40851_2015_22_MOESM1_ESM.doc]

**Table S1** Summary of study sites for collection of cicada exuviae.

| Site name | Code | Latitude(ºN) | Longitude(ºE) | City | Patch type | No. plots | Altitude (m) | Area (ha) |
| --- | --- | --- | --- | --- | --- | --- | --- | --- |
| Jonan Park | SPs | 34.68 | 135.53 | Osaka | small park | 1 | 3 | 0.54 |
| Saishoyama Park | SPs | 34.67 | 135.53 | Osaka | small park | 1 | 6 | 2.80 |
| Sanadayama Park | SPs | 34.67 | 135.53 | Osaka | small park | 1 | 12.5 | 3.04 |
| Karakiyomachi Park | SPs | 34.67 | 135.52 | Osaka | small park | 1 | 15 | 0.49 |
| Kozu Park | SPs | 34.67 | 135.51 | Osaka | small park | 1 | 21 | 1.64 |
| Ikutama Park | SPs | 34.66 | 135.51 | Osaka | small park | 1 | 7 | 3.74 |
| Osaka Castle Park | LP1 | 34.69 | 135.53 | Osaka | large park | 3 | 5.5-6 | 106.7 |
| Nagai Park | LP2 | 34.61 | 135.52 | Osaka | large park | 3 | 6-8 | 65.7 |
| Osaka City University | LP3 | 34.59 | 135.51 | Osaka | large park | 4 | 10.5-12 | 25.5 |
| Oizumi Park | LP4 | 34.57 | 135.53 | Sakai | large park | 3 | 21-22 | 96.7 |
| Mt. Hiraoka, lower site | F1 | 34.67 | 135.65 | Higashi-Osaka | forest | 4 | 73-91 | - |
| Mt. Hiraoka, higher site | F2 | 34.67 | 135.66 | Higashi-Osaka | forest | 6 | 291-319 | - |
| Mt. Mino | F3 | 34.84 | 135.47 | Mino | forest | 3 | 229-246 | - |
