# Supplementary material for: Urban soil compaction reduces cicada diversity
Source: Zoological Lett. 2015 Aug 1;1:19. doi: 10.1186/s40851-015-0022-3 (PMC4657352; doi:10.1186/s40851-015-0022-3)
Supplement: Additional file 2: Table S2. — Soil properties at representative sampling plots. [file 40851_2015_22_MOESM2_ESM.doc]

**Table S2** Soil properties at representative sampling plots.

| Site#plot ID^*^ | Soil hardness (mm)^†^ |  | Dry bulk density (g/ml) ^†^ |  | Water content (%)^†^ |  | Proportion of *C. facialis*^‡^ |
| --- | --- | --- | --- | --- | --- | --- | --- |
| LP3#1 | 18.21 ± 5.15 |  | 1.20 ± 0.11 |  | 6.58 ± 2.49 |  | 0.853 |
| LP3#2^§^ | 21.57 ± 3.96 |  | 1.35 ± 0.10 |  | 8.82 ± 2.89 |  | 1.000 |
| LP3#3 | 24.57 ± 3.37 |  | 1.40 ± 0.07 |  | 5.81 ± 1.69 |  | 1.000 |
| LP4#1 | 14.76 ± 3.77 |  | 1.06 ± 0.18 |  | 12.89 ±2.63 |  | 0.161 |
| F1#1 | 20.06 ± 4.80 |  | 1.23 ± 0.13 |  | 13.12 ± 2.55 |  | 0.632 |
| F1#2 | 18.84 ± 6.13 |  | 1.25 ± 0.11 |  | 14.73 ± 3.00 |  | 0.667 |
| F1#3 | 17.57 ± 4.93 |  | 0.89 ±0.10 |  | 20.56 ± 2.97 |  | 0.091 |
| F2#1 | 11.87 ± 4.21 |  | 0.85 ± 0.10 |  | 20.69 ± 3.24 |  | 0.000 |
| F2#5 | 11.12 ± 3.31 |  | 0.80 ± 0.19 |  | 15.66 ± 2.00 |  | 0.000 |
| F2#6 | 9.91 ± 3.57 |  | 0.54 ± 0.20 |  | 25.08 ± 5.99 |  | 0.000 |

*Site code names reflect patch types: LP means the large park, and F means the forest patch type.

^†^Values indicate mean ± SD.

^‡^Proportion of *Cryptotympana facialis* to all collected exuviae in the plot.

^§^The site of soil collection for burrowing experiments.
