# Supplementary material for: Urban soil compaction reduces cicada diversity
Source: Zoological Lett. 2015 Aug 1;1:19. doi: 10.1186/s40851-015-0022-3 (PMC4657352; doi:10.1186/s40851-015-0022-3)
Supplement: Additional file 3: Table S3. — Correlation table for the explanatory variables. [file 40851_2015_22_MOESM3_ESM.doc]

**Table S3** Correlation table for the explanatory variables.

|  | Imp | Alt | Slp | Shd | Can | Tdv | Tdn |
| --- | --- | --- | --- | --- | --- | --- | --- |
| Imperviousness (Imp) | - |  |  |  |  |  |  |
| Altitude (Alt) | **–0.92** | - |  |  |  |  |  |
| Slope (Slp) | **–**0.67 | 0.68 | - |  |  |  |  |
| Soil hardness (Shd) | **0.82** | **–0.81** | **–**0.64 | - |  |  |  |
| Canopy (Can) | **–0.75** | 0.62 | 0.39 | **–**0.70 | - |  |  |
| Tree diversity (Tdv) | 0.47 | **–**0.45 | -0.29 | 0.41 | **–**0.38 | - |  |
| Tree density (Tdn) | **–**0.35 | 0.24 | 0.09 | **–**0.29 | 0.38 | **–**0.45 | - |
